# Investigating the Mechanisms behind the Positive Food Effect of Abiraterone Acetate: In Vitro and Rat In Situ Studies

**DOI:** 10.3390/pharmaceutics14050952

**Published:** 2022-04-28

**Authors:** Marlies Braeckmans, Patrick Augustijns, Raf Mols, Cécile Servais, Joachim Brouwers

**Affiliations:** 1 Drug Delivery and Disposition, Department of Pharmaceutical and Pharmacological Sciences, KU Leuven, Gasthuisberg O&N II, Herestraat 49, P.O. Box 921, 3000 Leuven, Belgium; marlies.braeckmans@kuleuven.be (M.B.); raf.mols@kuleuven.be (R.M.); joachim.brouwers@kuleuven.be (J.B.); 2 Galephar M/F Research Center, 6900 Marche-en-Famenne, Belgium; cserv@galephar.be

**Keywords:** abiraterone acetate, solubility, permeability, AMI-system, rat in situ intestinal perfusion, food effect, lipid digestion

## Abstract

The anticancer agent abiraterone suffers from an extensive positive food effect after oral intake of the prodrug abiraterone acetate (Zytiga). The underlying processes determining postprandial abiraterone absorption were investigated in this study. The impact of lipids and lipid digestion products on (i) the solubility of abiraterone acetate and abiraterone, (ii) the conversion of abiraterone acetate to abiraterone, and (iii) the passive permeation of abiraterone was determined in vitro. The interaction of abiraterone acetate and abiraterone with vesicles and colloidal structures in the simulated fed state media containing undigested lipids and lipid digestion products enhanced the solubility of both compounds but limited the esterase-mediated hydrolysis of abiraterone acetate and the potential of abiraterone to permeate. Rat in situ intestinal perfusion experiments with a suspension of abiraterone acetate in static fed state simulated media identified abiraterone concentrations in the perfusate as the main driving force for absorption. However, experiments with ongoing lipolysis in the perfusate highlighted the importance of including lipid digestion as a dynamic process when studying postprandial abiraterone absorption. Future research may employ the in situ perfusion model to study postprandial drug absorption from a dynamic lipolysis-mediated intestinal environment to provide reference data for the optimisation of relevant in vitro models to evaluate food effects.

## 1. Introduction

The postprandial gastrointestinal environment is known to impact the absorption of (lipophilic) poorly water-soluble drugs. Not only does the gastrointestinal (GI) physiology change (e.g., prolonged gastric residence time and increased gastric pH) but also the composition of GI fluids changes continuously as food digests [1]. Lipids, their digestion products, bile salts, phospholipids and cholesterol associate to form a wide range of different colloidal structures, which change in composition, shape and size as digestion progresses [2,3]. Lipophilic drugs, in particular, are known to be better solubilised in fed state human intestinal fluids (FeHIF), which may or may not result in higher oral bioavailability. Abiraterone acetate (Zytiga), used for the treatment of prostate cancer, is an example of such a highly lipophilic drug (Log P 5.12) and is known for its extensive positive food effect [4,5]. Depending on the lipid content of the meal, its bioavailability increases 5- to 10-fold [6]. Abiraterone acetate is the ester prodrug of the active compound abiraterone, which suffers from poor solubility and oral bioavailability. Although the bioavailability of abiraterone is increased when formulated as the ester prodrug due to improved dissolution properties, abiraterone acetate is classified as a BCS IV drug [4]. Despite the increase in bioavailability when taken in the fed state, abiraterone acetate is advised to be taken on an empty stomach (i.e., at least 2 h after and 1 h before eating) to reduce the risk for side effects caused by variability in absorption. Due to the limited absorption in the fasted state, the dose is set at 1000 mg a day, which is largely excreted in the faeces [7].

The mechanisms underlying the intestinal absorption of abiraterone in the fed state after Zytiga administration remain unresolved despite extensive research. In the fasted state, rapid degradation of the ester prodrug results in supersaturated abiraterone concentrations in the duodenum of healthy volunteers after intake of 250 mg Zytiga [4], driving intestinal absorption. In the fed state, no supersaturation has been observed in vivo, probably due to the higher apparent solubility of abiraterone in FeHIF in addition to the slower dissolution and hydrolysis of abiraterone acetate. Overall, abiraterone (acetate) concentrations have been found to be lower in the fed state duodenum compared to the fasted state, which did not reflect the systemic absorption. More research is needed regarding the mechanism that drives the absorption in the fed state [5].

Therefore, the present study aimed to further investigate the underlying processes determining postprandial abiraterone absorption. The highly dynamic GI environment makes it difficult to simulate the in vivo situation using in vitro and/or in silico models. Current in vitro assays do not always consider the lipids present in FeHIF. For instance, when studying drug solubility in FeHIF, the upper lipid layer is usually discarded and solubilisation by lipid droplets and large lipid colloids (e.g., vesicles) is not considered. When using the total sample instead of only the micellar fraction, the solubilising capacity of FeHIF samples from 10 different volunteers was indeed enhanced for four lipophilic compounds [8]. Although drug solubility in the micellar layer is considered most important for absorption, it is unclear to what extent drug solubilised within the lipid phase contributes to the total absorptive flux. Evaluating the effect of food on drug absorption should therefore not be limited to solubility and dissolution testing but should also involve permeation testing from (simulated) postprandial media. However, cell-based permeation models are often not suited to assess the effect of food on drug absorption due to the increased complexity of the medium affecting monolayer integrity. Cell-free permeation models such as the artificial membrane insert system (AMI-system) provide a good alternative to study passive permeation as the majority of compounds are taken up via transcellular diffusion [9]. The most in vivo relevant method to study food effects remains using an intact intestinal barrier, which is present in the rat in situ perfusion method. The in situ perfusion technique in rats with mesenteric sampling is considered a valuable method to study drug absorption as the most important drug transporters and P450 enzymes are expressed in rat enterocytes [10]. Carboxylesterase expression in the small intestine makes the in situ perfusion method also suitable for studying the intestinal absorption of ester prodrugs. In addition, lipid digestion products might be absorbed, thereby changing the ultrastructure of the perfused medium and reflecting the dynamic in vivo situation more closely compared to, for instance, the AMI-system.

The aim of this research was to further identify the underlying mechanisms contributing to the positive food effect of abiraterone acetate. More specifically, the role of lipids and lipid degradation products in the uptake of abiraterone (acetate) was studied. The impact of lipids and lipid digestion products was determined in vitro on (i) the solubility of abiraterone acetate and abiraterone, (ii) the passive permeation of abiraterone using the AMI-system and (iii) the conversion of abiraterone acetate to abiraterone. In order to combine effects on dissolution, prodrug hydrolysis and transport across an intact small intestinal barrier, the absorption of abiraterone from an abiraterone acetate suspension in different media with varying lipid composition was studied using the in situ rat perfusion model with mesenteric blood sampling.

## 2. Materials and Methods

### 2.1. Chemicals

Abiraterone, abiraterone acetate and their deuterated standards abiraterone-d4 and abiraterone acetate-d4 were kindly provided by Janssen Research & Development (Beerse, Belgium). Esterase from porcine liver and pancreatin from porcine pancreas (8 × USP specification) were obtained from Sigma–Aldrich (St. Louis, MO, USA). Hanks’ Balanced Salt Solution (HBSS) and 4-(2-hydroxyethyl)-1-piperazineethanesulfonic acid (HEPES) were obtained from Lonza (Verviers, Belgium). Biorelevant (London, UK) supplied FaSSIF/FeSSIF/FaSSGF powder. Ketamine (Nimatek) and xylazine (Xyl-M 2%) were obtained from Dechra (Lille, Belgium) and VMD (Arendonk, Belgium), respectively. Methanol, acetonitrile and formic acid (99%) were purchased from Biosolve (Valkenswaard, The Netherlands). Fisher Scientific (Waltham, MA, USA) supplied glucose. Dimethyl sulfoxide (DMSO) was bought from Acros Organics (Geel, Belgium). Water was purified with a Maxima system (Elga Ltd., High Wycombe Bucks, UK). D-α-tocopheryl polyethylene glycol 1000 succinate (TPGS) was obtained from Eastman Chemical Company (Kingsport, TN). Regenerated cellulose membrane (molecular weight cutoff 2 kDa) and methyl-tert-butyl ether were purchased from VWR (Leuven, Belgium). Ensure Plus (proteins 6.25 g/100 mL, carbohydrates 20.20 g/100 mL and lipids 4.92 g/100 mL; Abbott Laboratories B.V., Zwolle, The Netherlands) was used to simulate a standard meal. Cream (35% fat, Boni) was purchased from Colruyt (Heverlee, Belgium). Glycerol monooleate and sodium oleate were obtained from Danisco A/S (Grindstad, Denmark) and TCI Chemicals Europe N.V. (Zwijndrecht, Belgium), respectively.

### 2.2. Media

Transport medium was prepared by dissolving 25 mM glucose in 500 mL HBSS, buffered with 10 mM HEPES and adjusted to pH 7.4 with 1M NaOH. TPGS-solution was obtained by dissolving TPGS (0.2% *w*/*v*) in transport medium. Fasted simulated intestinal fluid (FaSSIF) was prepared by dissolving 2.24 mg/mL FaSSIF/FeSSIF/FaSSGF powder in blank FaSSIF phosphate buffer (3.95 mg/mL NaH_2_PO_4_·H_2_O, 6.19 mg/mL NaCl, 0.42 mg/mL NaOH, adjusted to pH 6.5).

Different media were prepared to simulate the fed state. All fed state media were based on a modified version of fed state simulated intestinal fluid (FeSSIF), with the pH set to 6.5 instead of pH 5.0 to avoid pH related effects, further referred to as FeSSIF*. FeSSIF* was prepared by dissolving 11.2 mg/mL FaSSIF/FeSSIF/FaSSGF powder in blank FaSSIF phosphate buffer adjusted to pH 6.5. Two simulated fed state media were based on the nutrient shake Ensure Plus while four other media were prepared by adding fixed amounts of triglycerides (TG) and/or lipid digestion products to FeSSIF*.

#### 2.2.1. Addition of Undigested TG: FeSSIF*-T_h_ and FeSSIF*-E

Two simulated fed state media containing the same (high) amount of undigested TG (i.e., 8.2 mg/mL) from a different source were prepared. To obtain FeSSIF*-T_h_, cream (350 mg/mL TG) was diluted in FeSSIF* while FeSSIF*-E was prepared by adding 2 mL of Ensure Plus to 10 mL of FeSSIF*.

#### 2.2.2. Addition of Lipid Digestion Products: FeSSIF*-FM, FeSSIF*-FMT and FeSSIF*-FMT_h_

Glycerol monooleate and sodium oleate were used as monoglycerides (MG) and free fatty acids (FFA), respectively. Cream (35% fat) was used as the TG source. Concentrations of TG (0.91 mg/mL), MG (3.8 mg/mL) and FFA (8.8 mg/mL) were based on the work of Riethorst et al., representing the 75% percentile of FeHIF samples collected from 20 healthy volunteers [11]. A higher amount of TG was also added to resemble the lipid fraction of FeSSIF*-T_h_ and FeSSIF*-E (i.e., 8.2 mg/mL).

FeSSIF*-FM, containing FFA and MG, was prepared by adding 8.8 mg/mL sodium oleate and 3.8 mg/mL glycerol monooleate to FeSSIF*. FeSSIF*-FMT and FeSSIF*-FMT_h_ were prepared in the same way but with the addition of cream to obtain 0.91 mg/mL and 8.2 mg/mL TG, respectively.

#### 2.2.3. Inclusion of Lipid Digestion: FeSSIF*-E_d_

To evaluate the impact of ongoing lipolysis and include a more dynamic set-up in contrast to the static media, FeSSIF*-E was digested by adding pancreatin (0.83 mg/mL) 15 min prior to the start of the experiment without inhibition of lipolysis throughout the experiment (i.e., FeSSIF*-E_d_). The composition of all media is summarised in Table 1.

### 2.3. Solubility Measurements

The apparent solubility (i.e., including both molecules freely dissolved in the aqueous phase and molecules solubilised in colloidal structures and lipid droplets [12]) of abiraterone and abiraterone acetate was determined in the different simulated fed state media (i.e., FeSSIF*, FeSSIF*-FM, FeSSIF*-FMT, FeSSIF*-FMT_h_, FeSSIF*-T_h_ and FeSSIF*-E) using the shake flask method. Approximately 0.3 mg of compound was added to microcentrifuge tubes containing 0.3 mL of the abovementioned media and placed in a prewarmed shaking incubator (37 °C, 175 rpm; KS 4000 I control incubator from Ika (Staufen, Germany)) for 24 h. To the media containing lipid digestion products, 0.9 mg of compound was added instead of 0.3 mg. All solubility experiments were performed in triplicate. Afterwards, samples were centrifuged to separate the solid phase from the dissolved part (20.817 g, 30 min at 37 °C). In the media containing TG, drug solubility was measured in (i) the lower layer of the supernatant (i.e., the micellar layer) and (ii) the full supernatant, including both the micellar and lipid layer. To determine the solubility in the micellar layer, the top lipid layer was aspirated upon which the supernatant was diluted in MeOH:H_2_O (50:50 *v*/*v*) containing IS (abiraterone-d4 and abiraterone acetate-d4, final concentration 50 nM). To determine the solubility in the total sample, the entire supernatant was transferred into a new microcentrifuge tube and vortexed upon which the supernatant was diluted in MeOH:H_2_O (50:50 *v*/*v*) containing IS to fit the linear range.

### 2.4. Enzymatic Conversion Experiments

The conversion of abiraterone acetate to abiraterone was studied in the abovementioned media using either 80 IU/mL esterase from porcine liver or 0.83 mg/mL porcine pancreatin. A total of 150 µM abiraterone acetate (DMSO stock solution) was added to 2 mL of the media in a 12-well plate and placed in a prewarmed shaking incubator (37 °C, 300 rpm; Thermostar, BMG Labtech, Offenburg, Germany). Samples were obtained in triplicate at predetermined time points and centrifuged immediately (20.817 g, 5 min). When lipids were present, drug concentrations were determined in both total (lipid + micellar) and micellar layer as described above.

### 2.5. Permeability Assessment Using the AMI-System

To investigate the impact of TG and lipid digestion products (FFA, MG) on the passive permeation of abiraterone, transport experiments were performed in the AMI-system according to Berben et al. [13]. In short, a regenerated cellulose membrane (molecular weight cut-off 2 kDa) was mounted between two plastic rings resulting in a surface area of 4.91 cm^2^. Membrane integrity was checked by water leakage. The AMI-system was placed in a six-well plate on a shaking incubator at 37 °C and 300 rpm. A 1:3 ratio was maintained between donor and acceptor volumes by applying 2 mL of TPGS-solution to the acceptor compartment and 665 µL of the donor solution of abiraterone in the media prepared as described above. The initial concentration of abiraterone depended on the medium to ensure donor concentrations below the solubility and sufficient permeation to quantify the permeated amount in the acceptor compartment: 10 μM in FaSSIF, 30 μM in FeSSIF*, FeSSIF*-T_h_ and FeSSIF*-E, and 150 μM in FeSSIF*-FM, FeSSIF*-FMT and FeSSIF*-FMT_h_. Samples (100 µL) were taken from the acceptor compartment at 15, 30, 45, 60 and 120 min and diluted 1:1 in MeOH:H_2_O (50:50 *v*/*v*) containing IS. Subsequently, the withdrawn volumes were replaced by fresh TPGS-solution.

The apparent permeability coefficient (*P_app_*) was calculated from the cumulative amount of abiraterone appearing in the acceptor compartment according to Equation (1):

(1) $$P_{app}=\frac{dQ}{dt}\times \frac{1}{A\times C_{donor}}$$

where *dQ*/*dt* is the amount of abiraterone appearing in the acceptor compartment over time, *A* represents the effective surface area available for transport, and *C_donor_* is the initial concentration in the donor compartment. Data are represented as mean ± standard deviation (SD).

### 2.6. Absorptive Flux Assessment in Rats

Purpose-bred, male Wistar rats (Janvier, Le Genest Saint-Isle, France) were used for the in situ intestinal perfusion experiments. Rats were housed according to the Belgian and European laws, guidelines and policies for animal experiments, in the Central Animal Facilities of the university. This project was approved by the Institutional Ethical Committee for Animal Experimentation (P144/2016, approval date 1/10/2016).

#### In Situ Intestinal Perfusion

Rat in situ intestinal perfusion experiments were performed according to Stappaerts et al. [4]. In short, rats of approximately 300–350 g were anaesthetised using a mixture of ketamine (87.5 mg/kg) and xylazine (0.875 mg/kg). Blood from donor rats was given via cannulation of the right jugular vein with a heparinised (50 IU/mL) polyethylene cannula (o.d. 1.02 mm; Portex, Kent, UK). After a laparotomy, a distal segment of the small intestine (ileum) was isolated by inserting two glass cannulas (o.d. 4 mm, i.d. 3 mm) at the proximal and distal end of the segment. After removal of intestinal contents using transport medium, the intestinal segment was preincubated with FaSSIF or FeSSIF* for 30 min at a perfusion flow rate of 1 mL/min. After 30 min, the mesenteric vein draining the isolated part of the intestinal segment was cannulated using the top end (1 cm) of a catheter (Insyte-W 0.7 × 19 mm; Becton Dickinson, Salt Lake City, UT, USA) and a 1-hour incubation was started, using a suspension of abiraterone acetate as perfusion medium at 1 mL/min. This suspension was made while preparing and preincubating the rat intestine. First, esterases (80 IU/ml) or pancreatin (0.83 mg/mL) were added to one of the above-mentioned media (i.e., FaSSIF, FeSSIF*, FeSSIF*-FM, FeSSIF*-FMT, FeSSIF*-FMT_h_, FeSSIF*-T_h_, FeSSIF*-E or FeSSIF*-E_d_) and stirred at 100 rpm and 37 °C for 15 min. Next, 6 mL of this mixture was added to a test tube containing 3 mg abiraterone acetate. The resulting suspension was placed in a water bath at 37 °C and stirred at 300 rpm for 30 min before the start of the incubation. During the incubation, blood was collected over 5-min intervals. All in situ perfusion experiments were performed in the closed loop set-up to avoid excessive use of medium and compound. Donor blood was supplied via the jugular vein at a rate of 0.3 mL/min using a syringe pump (Pilot A2, Fresenius Vial, Grenoble, France) to maintain hemodynamic conditions. To verify donor concentrations, perfusate samples were taken every 10 min followed by a centrifugation step (5 min, 20.817 g, room temperature). The supernatant was diluted with MeOH:H_2_O (50:50 *v*/*v*) containing IS to fit the linear range. When lipids were present in the medium, drug concentrations were determined in both total (lipid + micellar) and micellar layer as described above.

The absorptive flux of abiraterone was determined from the slope of the cumulative amount of abiraterone appearing in the mesenteric blood at steady state according to Equation (2):

(2) $$Flux=\frac{dQ}{dt}\times A$$

where *dQ/dt* is the amount of compound appearing in the mesenteric blood over time and *A* is the surface area of the perfused intestinal segment. A mean intestinal segment radius of 0.2 cm was used. Data are presented as mean ± SD. The Pearson’s correlation coefficient was used to quantify the correlation between the abiraterone AUC_0–60min_ in the perfusate and the absorptive flux.

### 2.7. Analysis

Samples were analysed using an Acquity H-class UPLC system (Waters, Milford, MA, USA), equipped with a Kinetex XB—C18 column (2.6 μm, 2.1 × 50 mm; Phenomenex, Utrecht, The Netherlands) held at 35 °C. The mobile phase consisted of a mixture of methanol (solvent A) and water (solvent B) at a flow rate of 500 µL·min^−1^. Gradient elution was performed as follows: 40% of solvent A during 0.5 min, followed by 95% for 2.5 min. After 4 min, the column was rinsed with ACN:H_2_O (90:10 *v*/*v*) for 2 min, after which the column was re-equilibrated with starting conditions during 3 min. Abiraterone(-d4) and abiraterone acetate(-d4) eluted after 2.37 min and 2.64 min, respectively. The injection volume was 0.1 μL.

The mass spectrometer was operated in MS/MS positive ionisation mode with following parameters: source temperature 150 °C; capillary voltage 2.5 kV; cone voltage 60 V; cone flow 20 L N_2_/h; desolvation gas flow 800 L N_2_/h; desolvation temperature 600 °C. The mass transitions used for the detection of different compounds were m/z abiraterone 350.30 → 156.11 (collision energy: 52 V), *m*/*z* abiraterone-d4 354.32 → 159.10 (collision energy: 50 V), abiraterone acetate 392.30 → 332.30 (collision energy: 32 V) and m/z abiraterone acetate-d4 396.37 → 336.34 (collision energy: 30 V) with a dwell time of 35 ms for all compounds. Calibration curves were made in MeOH:H_2_O (50:50 *v*/*v*) and were linear between 500 nM and 1 nM. Quality control samples were prepared in MeOH:H_2_O (50:50 *v*/*v*) containing 500, 50 and 5 nM abiraterone (acetate) and were included on the days of analysis. Precision and accuracy errors were less than 15%.

For the analysis of the mesenteric blood samples from the in situ rat perfusion experiments, blood samples were centrifuged at the end of the experiment (4 °C, 2.880 g, 10 min; Centrifuge 5804R, Eppendorf, Hamburg, Germany). Subsequently, plasma was stored at −20 °C pending analysis. To quantify abiraterone concentrations, 400 µL of ACN:H_2_O (50:50 *v*/*v*), 100 µL of IS (final concentration 75 nM abiraterone-d4 in MeOH:H_2_O (70:30 *v*/*v*)) and 200 µL of NaHCO_3_ (5% *w*/*v*) were added to 100 µL of plasma and vortexed for 10 s. After extraction with 2 mL methyl tert butylether (vortex for 1 min) and centrifugation (2.880 g, 10 min, 4 °C), the organic layer was transferred to a clean test tube and evaporated under a gentle stream of air. Residue was redissolved in 150 μL of MeOH:H_2_O (70:30 *v*/*v*) and centrifuged (5 min, 20.817 g, room temperature) before injection into the LC–MS/MS. The same HPLC–MS/MS method was used as described above. Calibration curves were made by spiking blank plasma and were linear between 1.5 μM and 1.5 nM. Precision and accuracy errors were less than 15%.

## 3. Results and Discussion

To gain more insight into the underlying mechanism(s) of the positive food effect on abiraterone absorption following oral intake of the prodrug abiraterone acetate, solubility of abiraterone acetate and abiraterone, passive permeability of abiraterone and prodrug hydrolysis were studied in vitro, using different media reflecting the fed state. Although the commercially available media FeSSIF and FeSSIF-v2 reflect the average in vivo situation regarding postprandial concentrations of bile salts and phospholipids, lipids are not included. FeSSIF-v2 does include lipid digestion products (i.e., 0.8 mM oleate and 5 mM glycerol monooleate) but no undigested lipids constituting larger structures such as vesicles and lipid droplets, which also contribute to the solubilisation of lipophilic compounds [8]. Therefore, four different simulated media reflecting the fed state were prepared using TG and/or lipid digestion products in order to increase the in vivo relevance. In addition, two fed state media were based on the nutrient shake Ensure Plus, which is commonly used during in vivo aspiration studies to simulate the fed state. All media are summarised in Table 1. The pH of all media was set to 6.5 to avoid pH related effects.

### 3.1. Solubility

The solubility of abiraterone acetate and abiraterone was determined in the different media reflecting the fed state (Table 2). The solubility of both compounds was higher in FeSSIF* compared to FaSSIF. The addition of FFA and MG to FeSSIF* (i.e., FeSSIF*-FM) greatly increased the solubilising capacity, probably due to the formation of (mixed-) micelles. The solubility of abiraterone acetate and abiraterone increased 14-fold and 5-fold in FeSSIF*-FM compared to FeSSIF*, respectively.

In the media containing TG (i.e., FeSSIF*-FMT, FeSSIF*-FMT_h_, FeSSIF*-T_h_, and FeSSIF*-E), an upper lipid layer was present after centrifugation. Therefore, the solubility of abiraterone acetate and abiraterone was determined in the total sample (i.e., including the lipid layer) and in the micellar layer. The addition of TG to the media containing lipid digestion products (i.e., FeSSIF*-FMT, FeSSIF*-FMT_h_) resulted in superior solubility of abiraterone acetate. The solubility of abiraterone increased as well, but to a lesser extent. Abiraterone acetate (Log P 5.12) displayed higher affinity for the lipid layer compared to abiraterone (Log P 3.97), most likely due to its higher lipophilicity. Abiraterone acetate concentrations in the total sample increased 2-fold in FeSSIF*-FMT_h_ compared to FeSSIF*-FMT.

Adding TG to FeSSIF* (i.e., FeSSIF*-T_h_ and FeSSIF*-E) only slightly increased the solubilising capacity for abiraterone. The solubility of abiraterone acetate increased in the micellar layer although to a lesser extent compared to the media containing lipid digestion products. Abiraterone acetate solubility was further enhanced in the total sample, confirming the higher affinity of abiraterone acetate for the lipid layer.

Based on these results, it can be stated that lipid digestion products (MG and FFA) greatly increase the solubilising capacity of fed state media for both abiraterone acetate and abiraterone. The presence of TG has additional impact on the solubilisation of especially abiraterone acetate.

### 3.2. Passive Permeation (AMI-System)

**Figure 1:**
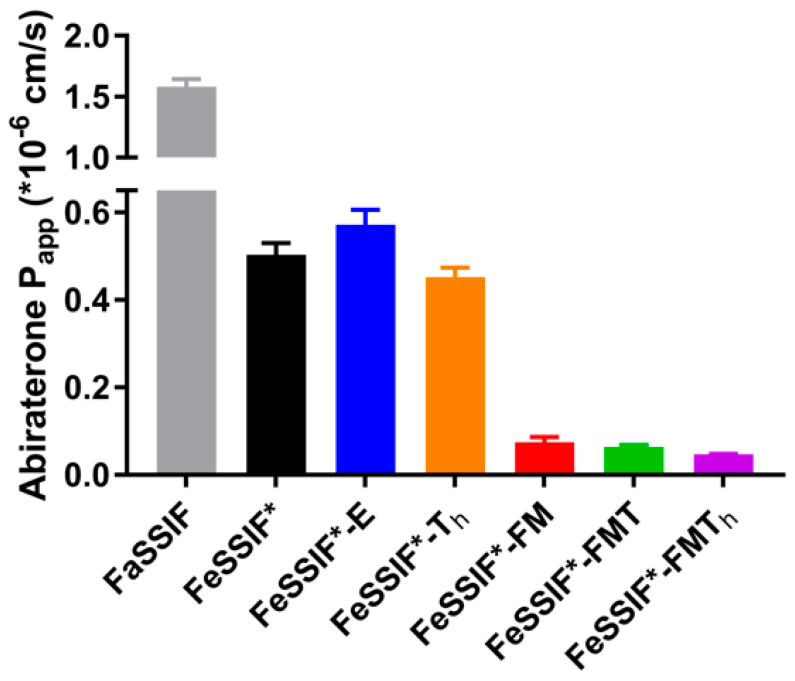
Apparent permeability coefficients (*P_app_*) of the AMI-system for abiraterone (solution) in FaSSIF and different simulated media reflecting the fed state (F = fatty acids, M = monoglycerides, T = triglycerides, T_h_ = high triglycerides, E = Ensure Plus). Data are shown as mean ± SD (n = 3).

The interaction of a drug with lipids and lipid digestion products may not only influence its apparent solubility, but also its apparent permeability. The AMI-system was chosen as a suitable in vitro model to study the impact of lipids and lipid digestion products on the passive permeation of abiraterone from a solution (Figure 1). Only abiraterone was used in these experiments as abiraterone acetate permeation across the cellulose membrane was too low to assess the effect of lipids and lipid digestion products.

In comparison to the *P_app_* values obtained for 14 compounds in FaSSIF and FaHIF by Berben et al. [13], abiraterone displayed low permeability in FaSSIF, as was expected due to it being a BCS class IV compound [4]. The *P_app_* value further decreased in the different fed state media. The higher bile salt and phospholipid concentrations in FeSSIF* compared to FaSSIF lowered the permeability for abiraterone 2.6-fold. The addition of TG to FeSSIF* (i.e., FeSSIF*-T_h_ and FeSSIF*-E) did not have an effect on the *P_app_*, probably due to the limited affinity of abiraterone for undigested lipids, which was observed in the solubility data (Table 2). In contrast, the extensive solubilisation of abiraterone within the colloidal structures formed by the addition of FFA and MG (Table 2) led to a significant decrease in passive permeability when using FeSSIF*-FM, FeSSIF*-FMT and FeSSIF*-FMT_h_.

These results indicate encapsulation of abiraterone in (mixed-)micelles, hampering permeation. It should be noted that the significance of abiraterone encapsulation in the dynamic in vivo situation is difficult to predict using the AMI-system, as colloidal structures continuously change upon intestinal transit and absorption of lipid digestion products.

### 3.3. Abiraterone Acetate Hydrolysis

#### 3.3.1. Hydrolysis in FaSSIF and Simulated Fed State Media

**Figure 2:**
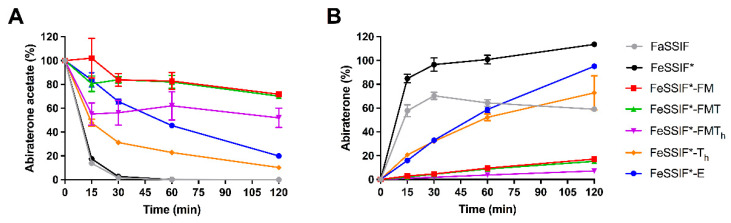
Concentration-time profiles of the degradation in abiraterone acetate (**A**) and the corresponding formation of abiraterone (**B**) in FaSSIF and fed state simulated media supplemented with porcine esterases (80 IU/mL). Concentrations are given as percentage of the molar start concentration of abiraterone acetate. In the media containing lipids, concentrations in the total sample (i.e., micellar + lipid layer) are presented. Data represent mean ± SD (n = 3). (F = fatty acids, M = monoglycerides, T = triglycerides, T_h_ = high triglycerides, E = Ensure Plus).

Esterases present in the small intestine hydrolyse abiraterone acetate to abiraterone in vivo. Hence, porcine esterases were used to simulate abiraterone acetate degradation and to evaluate how this degradation is affected by postprandial media. In FaSSIF and FeSSIF*, abiraterone acetate degraded relatively fast to abiraterone, with an almost full conversion within 30 min (Figure 2). In FaSSIF, a decrease in abiraterone concentration was observed starting from 60 min (Figure 2B), indicating abiraterone precipitation due to the low solubilising capacity of FaSSIF and the lack of an absorptive sink. In the media supplemented with undigested TG (i.e., FeSSIF*-T_h_ and FeSSIF*-E), slower degradation was observed. The interaction of abiraterone acetate with large lipid colloids, as indicated by the solubility data in Table 2, might hamper abiraterone acetate conversion by the esterases. In the media containing lipid degradation products (i.e., FeSSIF*-FM, FeSSIF*-FMT and FeSSIF*-FMT_h_), the conversion was very limited, in line with the superior solubilisation of abiraterone acetate in these media (Table 2). Overall, these results indicate superior stability of the ester prodrug in the lipolysis-mediated fed state, which was also observed in vivo by Geboers et al. [5]

#### 3.3.2. Pancreatin vs. Esterases

**Figure 3:**
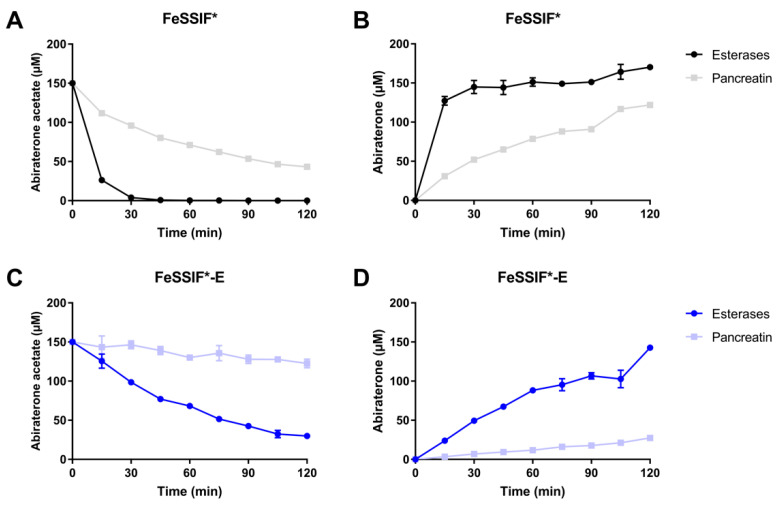
Concentration-time profile of the degradation study of abiraterone acetate in (**A**,**B**) FeSSIF* and (**C**,**D**) FeSSIF*-E supplemented with porcine esterases (80 IU/mL) or porcine pancreatin extract (0.83 mg/mL) after the addition of 150 μM abiraterone acetate and the corresponding formation of abiraterone (**B**,**D**). Results are expressed as mean ± SD (n = 3).

In the rat in situ experiments (see Section 3.4), porcine pancreatin was used to digest FeSSIF*-E. Besides different lipases, porcine pancreatin also contains esterases and, as such, is able to hydrolyse abiraterone acetate. To compare the hydrolysis of abiraterone acetate between porcine esterases and porcine pancreatin, a solution of abiraterone acetate was incubated in FeSSIF* and FeSSIF*-E with esterases or pancreatin. Abiraterone acetate degraded faster in FeSSIF* compared to FeSSIF*-E, regardless of the enzyme source (Figure 3). The esterase activity was lower in the experiments with pancreatin compared to porcine esterases. The hydrolysis of abiraterone acetate by pancreatin was not investigated in the media containing lipid digestion products as these media were designed to avoid the complex lipolysis process.

### 3.4. In Situ Rat Perfusion

The results described above indicate multiple effects of the interaction of abiraterone acetate and abiraterone with colloidal structures in simulated fed state media containing lipid digestion products. The interaction may not only enhance the solubility of both compounds, but also limit the esterase-mediated hydrolysis of abiraterone acetate and the potential of abiraterone to permeate. The presence of undigested TG increased the solubilising capacity for abiraterone acetate but not for abiraterone. All these effects may contribute to the in vivo food effect.

The in situ rat perfusion model allowed to simultaneously assess (i) the dissolution and solubilisation of abiraterone acetate, (ii) the esterase-mediated degradation to abiraterone, and (iii) the absorptive flux towards the mesenteric blood. Experiments were performed by perfusing abiraterone acetate suspended in FaSSIF or in the simulated media reflecting the fed state (Table 1) containing esterases. In addition to the static simulated fed state media, which were prepared by adding fixed amounts of TG, MG, FFA or Ensure Plus to FeSSIF* (i.e., FeSSIF*-FM, FeSSIF*-FMT, FeSSIF*-FMT_h_, FeSSIF*-T_h_ and FeSSIF*-E), an effort was made to increase the dynamic nature of the perfusate by including lipid digestion. Therefore, pancreatin was added to FeSSIF*-E instead of esterases, resulting in FeSSIF*-E_d_.

**Figure 4:**
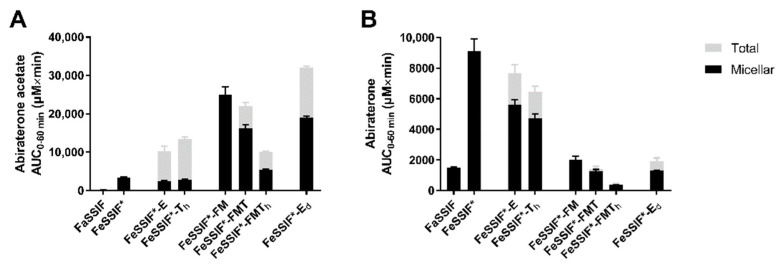
Average AUC_0–60min_ (± SD) of the total (grey) and micellar (black) perfusate concentration-time profiles of (**A**) abiraterone acetate and (**B**) abiraterone after rat intestinal perfusion of an abiraterone acetate suspension (0.5 mg/mL) in FaSSIF and in simulated fed state media. All media were supplemented with 80 IU/mL esterases, except for FeSSIF*-E_d_, in which pancreatin (0.83 mg/mL) was used. Results represent mean ± SD (n = 3; F = fatty acids, M = monoglycerides, T = triglycerides, T_h_ = high triglycerides, E = Ensure Plus, E_d_ = digested Ensure Plus).

**Figure 5:**
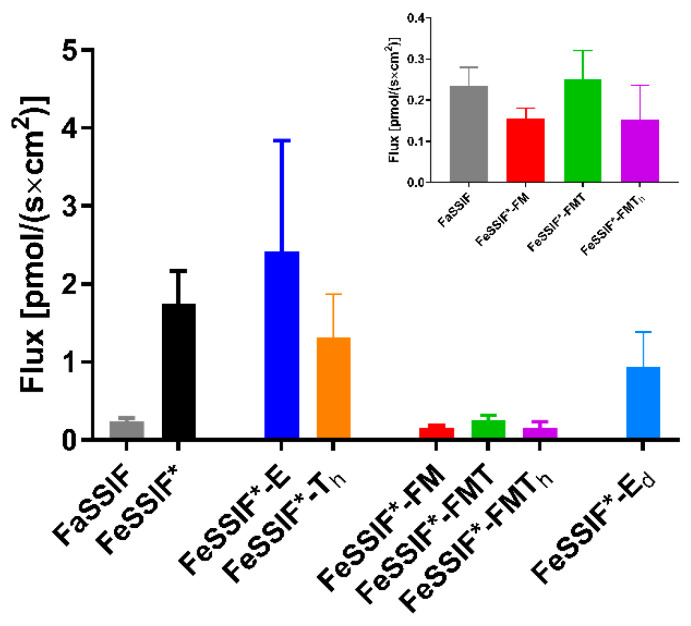
Absorptive flux values of abiraterone after rat intestinal perfusion of an abiraterone acetate suspension (0.5 mg/mL) in FaSSIF and in simulated fed state media. All media were supplemented with 80 IU/mL, except for FeSSIF*-E_d_, in which pancreatin (0.83 mg/mL) was used. Insert depicts the absorptive flux values of abiraterone in FaSSIF and media containing fixed amounts of lipid digestion products. Data represent mean ± SD (n = 3; F = fatty acids, M = monoglycerides, T = triglycerides, T_h_ = high triglycerides, E = Ensure Plus, E_d_ = digested Ensure Plus).

During the in situ experiments, the perfusate was sampled at the inlet of the intestinal segment to determine abiraterone (acetate) concentrations over the course of the experiment. The AUC_0–60min_ of the perfusate concentration-time profiles of abiraterone acetate and abiraterone are depicted in Figure 4. The corresponding abiraterone flux into the mesenteric blood is given in Figure 5.

#### 3.4.1. FaSSIF vs. FeSSIF*

**Figure 6:**
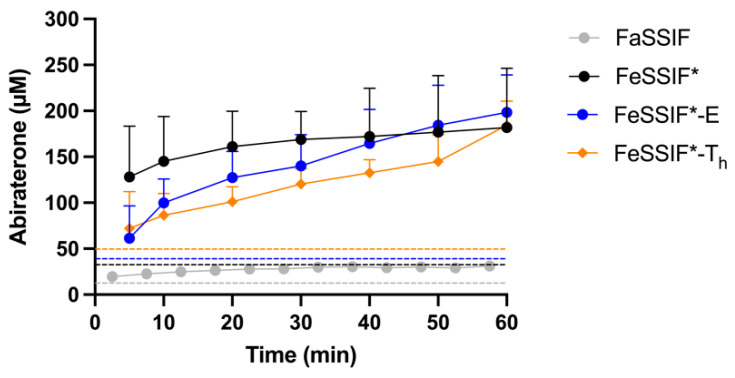
Abiraterone concentration-time profiles in FaSSIF, FeSSIF*, FeSSIF*-E (total) and FeSSIF*-T_h_ (total) during rat intestinal perfusion of an abiraterone acetate suspension (0.5 mg/mL). Horizontal colored dotted lines represent the (total) apparent solubility of abiraterone in the corresponding medium, indicating abiraterone supersaturation over the course of the experiment in all media. Data represent mean ± SD (n = 3).

As esterases were present in the media, the measured abiraterone acetate concentrations reflect the dissolution and hydrolysis of the prodrug. In vitro, prodrug hydrolysis was observed to depend on the medium (Figure 2). Although abiraterone acetate degraded at a similar rate in FaSSIF and FeSSIF* (Figure 2), the abiraterone AUC_0–60min_ in the perfusate was much higher in FeSSIF* compared to FaSSIF (Figure 4). This can be attributed to the higher solubilising capacity of FeSSIF* for both compounds. The fast turnover of abiraterone acetate by the high amount of esterases present in the perfusate generated apparent supersaturation of abiraterone in FaSSIF and FeSSIF* over the entire course of the experiment, as can be observed in the abiraterone concentration-time profiles in Figure 6. The high abiraterone concentrations in FeSSIF* compared to FaSSIF (Figure 4B andFigure 6) resulted in an increased abiraterone flux towards the mesenteric vein (Figure 5).

#### 3.4.2. The Effect of Undigested TG

The addition of undigested TG to FeSSIF* (i.e., FeSSIF*-E and FeSSIF*-T_h_) resulted in higher total abiraterone acetate concentrations compared to FeSSIF* (Figure 4A) due to the increased solubilisation (Table 2) and higher affinity of abiraterone acetate for the lipid layer. The slower prodrug hydrolysis (Figure 2) in these media generated slightly lower abiraterone concentrations compared to FeSSIF* (Figure 4B), although supersaturation was still observed (Figure 6). The apparent abiraterone supersaturation increased the absorptive flux in FeSSIF*-E and FeSSIF*-T_h_ compared to FaSSIF. The absorptive flux was similar between FeSSIF, FeSSIF*-E and FeSSIF*-T_h_, which was in line with the high abiraterone concentrations observed in the perfusate (Figure 4B andFigure 6).

#### 3.4.3. The Effect of Lipid Digestion Products

As the in vivo intestinal fed state environment is lipolysis mediated, FFA and MG were added to FeSSIF* in a fixed amount, with different amounts of TG (i.e., FeSSIF*-FM, FeSSIF*-FMT, FeSSIF*-FMT_h_). Abiraterone acetate concentrations remained high due to the high solubility (Table 2) and limited hydrolysis (Figure 2). The observed stability of abiraterone acetate resulted in low abiraterone concentrations in the perfusate (Figure 4B) and together with the poor passive permeability of abiraterone in these media (Figure 1) generated a low flux (Figure 5). The flux was comparable to the flux in FaSSIF, as similar abiraterone concentrations were measured in the perfusate. No supersaturation was observed (concentration-time profiles not shown).

**Figure 7:**
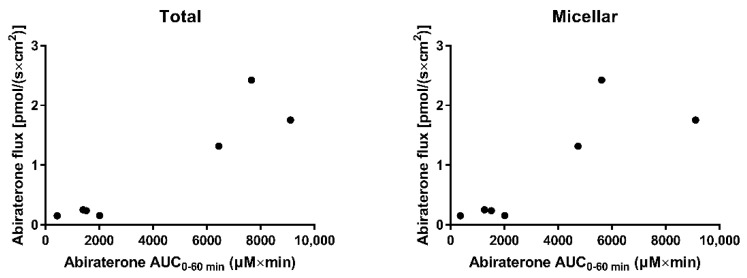
Correlation between the abiraterone flux towards the mesenteric vein and abiraterone AUC_0–60min_ obtained from the concentration-time profiles of the perfusate in FaSSIF, FeSSIF*, FeSSIF*-FM, FeSSIF*-FMT, FeSSIF*-FMT_h_, FeSSIF*-T_h_ and FeSSIF*-E. **Left**: total concentrations, **right**: micellar concentrations.

When considering the in situ perfusion data from all above-mentioned media, the absorptive flux correlated with the abiraterone AUC_0–60min_ of the concentration-time profiles from the perfusate, resulting in a Pearson correlation coefficient (r) of 0.9334 (*p* = 0.002) and 0.8460 (*p* = 0.0164) for the total and micellar AUC_0–60min_, respectively (Figure 7). No correlation could be observed between intraluminal abiraterone acetate concentrations and the abiraterone flux. Although it cannot be completely ruled out that abiraterone acetate permeates the intestinal wall followed by hydrolysis within the enterocytes, these data suggest that abiraterone is the main driving force for absorption in fed state conditions, requiring intraluminal abiraterone acetate hydrolysis.

#### 3.4.4. The Effect of Ongoing Lipolysis

When considering the in situ perfusion data described so far, a positive food effect on the absorptive flux of abiraterone (compared to FaSSIF) was observed in FeSSIF* and in the media in which undigested TG were added to FeSSIF* (i.e., FeSSIF*-E and FeSSIF*-T_h_). Even though a strong positive food effect is also seen on the absorption of abiraterone in vivo, the composition of these media does not represent the composition of fed state intestinal fluids in vivo, as no lipid digestion products are included. In addition, the high abiraterone concentrations obtained in those media (Figure 4B) are not representative for the low abiraterone concentrations observed in the fed state duodenum of healthy volunteers [5].

To increase the in vivo relevance of the simulated fed state media, lipid digestion products were added. For instance, the composition of FeSSIF*-FMT was based on FeHIF data collected from healthy volunteers regarding concentrations of TG and lipid degradation products. The perfusate data in FeSSIF*-FMT (Figure 4B), in which low abiraterone concentrations were measured, correspond to the low abiraterone concentrations measured in the fed state duodenum in vivo [5]. However, the absorptive flux, which was greatly enhanced in the fed state in vivo despite these low intraluminal abiraterone concentrations, was not increased in the rat in situ experiments with lipid digestion products (Figure 5). Overall, increasing the biorelevance of the composition of simulated fed state media by adding fixed amounts of lipid digestion products did not allow simulation of the positive food effect on the absorption of abiraterone.

In an attempt to further increase the in vivo relevance of the in situ set-up, the liquid meal used during the in vivo studies (i.e., Ensure Plus) was used as the TG source and digested over the course of the experiment using pancreatin (i.e., FeSSIF*-E_d_). No additional esterases were added to this medium as pancreatin was observed to hydrolyse abiraterone acetate (Figure 3). Because of the ongoing lipolysis, the composition of this medium evolves as a function of time and can be considered more dynamic compared to the previously used static media in which fixed amounts of FFA, MG and/or TG were added to FeSSIF*.

Due to the ongoing lipolysis, no equilibrium solubility of abiraterone (acetate) could be determined in FeSSIF*-E_d_. Based on the increased solubility of both compounds in the other media containing lipolysis products (i.e., FeSSIF*-FM, FeSSIF*-FMT, FeSSIF*-FMT_h_), the overall solubility of both compounds was expected to be higher in FeSSIF*-E_d_ compared to FeSSIF*-E, which only contains undigested Ensure Plus. Abiraterone acetate concentrations in the perfusate were indeed high (Figure 4A), presumably reflecting the higher solubilising capacity of the medium during lipolysis. The abiraterone acetate concentrations were in line with those obtained in FeSSIF*-FMT. Abiraterone concentrations were relatively low and similar to the concentrations in FaSSIF and FeSSIF*-FMT (Figure 4B) due to the increased prodrug stability in the presence of lipid digestion products (Figure 2). Nevertheless, the absorptive flux was substantially higher compared to FaSSIF and FeSSIF*-FMT (Figure 5). The lower flux observed in FeSSIF*-E_d_ compared to FeSSIF* might result from the lower esterase activity in pancreatin as observed in Figure 3 and the increased prodrug stability due to the presence of lipid digestion products.

The increased absorptive flux despite relatively low abiraterone concentrations in the perfusate obtained when using FeSSIF*-E_d_ as perfusion medium are in line with the observations previously made in vivo [5]. These results highlight the importance of including lipid digestion as a dynamic process, rather than using static fed state media, when aiming to simulate the positive food effect on the absorption of abiraterone. More research is needed to clarify the reasons why the lipolysis process is so critical. Different colloidal structures may be formed when digesting TG by pancreatin (in FeSSIF*-E_d_) compared to adding fixed amounts of lipid digestion products (in, for instance, FeSSIF*-FMT). Such differences could impact the potential of abiraterone to permeate, despite similar apparent concentrations. In addition, the continuously changing environment during lipolysis may cause a more dynamic equilibrium between free and solubilised drug molecules. This might be important in releasing solubilised abiraterone acetate and abiraterone for hydrolysis and absorption, respectively. Additional research should therefore focus on characterising the dynamic composition and ultrastructure of fed state media during food digestion in relation to solubilisation and hydrolysis of abiraterone acetate and permeation of abiraterone.

## 4. Conclusions

The mechanisms behind the pronounced positive food effect of abiraterone acetate have not been elucidated despite extensive research. Therefore, the effects of different fed state simulated media on processes determining abiraterone absorption were first evaluated in vitro. Undigested lipids and especially lipid digestion products enhanced abiraterone (acetate) solubility but limited the esterase-mediated hydrolysis of abiraterone acetate and the apparent permeability for abiraterone. These different processes were then combined in the rat in situ perfusion model to evaluate the overall impact of fed state media on abiraterone absorption. When using static fed state media, abiraterone concentrations in the perfusate appeared to be the main driving force for absorption. However, only by including lipid digestion as an ongoing process during the in situ perfusion experiments, an enhanced abiraterone flux compared to the fasted state was observed despite the generation of relatively low abiraterone concentrations in the perfusate, which is in line with the in vivo situation. As such, the study warrants more research on the integration of lipid digestion as a dynamic process in the evaluation of food effects on the absorption of lipophilic drugs. In this respect, the in situ perfusion model can be considered an adequate reference model for studying postprandial drug absorption as it allows the investigation of multiple processes underlying drug absorption in relation to the dynamic lipid digestion process. Data obtained with this technique may contribute to the optimisation of more dynamic in vitro models to study food effects.

## Figures and Tables

**Table 1:** Composition of FaSSIF and simulated media reflecting the fed state used for in vitro and in situ experiments.

Medium	FaSSIF/FeSSIF/FaSSGF Powder (mg/mL)	FFA (mg/mL)	MG (mg/mL)	TG (mg/mL)		Enzymes (mg/mL)
		Sodium Oleate	Glycerol Monooleate	Cream ^1^	Ensure Plus ^2^	Pancreatin
FaSSIF	2.24					
FeSSIF*	11.2					
FeSSIF*-FM	11.2	8.8	3.8			
FeSSIF*-FMT	11.2	8.8	3.8	0.91		
FeSSIF*-FMT_h_	11.2	8.8	3.8	8.2		
FeSSIF*-T_h_	11.2			8.2		
FeSSIF*-E	9.33				8.2	
FeSSIF*-E_d_	9.33				8.2	0.83 ^3^

F = fatty acids, M = monoglycerides, T = triglycerides, T_h_ = high triglycerides, E = Ensure Plus, E_d_ = digested Ensure Plus. ^1^ Cream: 350 mg/mL TG, ^2^ Ensure Plus: 42.9 mg/mL TG, ^3^ Added 15 min prior to the start of the experiment.

**Table 2:** Apparent solubility of abiraterone acetate and abiraterone in simulated media reflecting the fed state (pH 6.5). In case a lipid layer was present, solubility was determined in both the total sample (micellar + lipid layer) and micellar layer. Results are expressed as mean ± SD (n = 3).

Medium	Abiraterone Acetate (µM)		Abiraterone (µM)	
	Micellar	Total	Micellar	Total
FaSSIF	64.6 (±5.2) ^1^	n/a	12.7 (±4.2) ^1^	n/a
FeSSIF*	148 (±15.7)	n/a	32.7 (±1.34)	n/a
FeSSIF*-FM	2111 (±63)	n/a	176 (±8.63)	n/a
FeSSIF*-FMT	2018 (±114)	2284 (±103)	161 (±7.83)	199 (±13.6)
FeSSIF*-FMT_h_	1283 (±53.4)	4615 (±421)	158 (±13.0)	221 (±13.5)
FeSSIF*-T_h_	330 (±18.0)	947 (±144)	31 (±2.55)	50 (±2.01)
FeSSIF*-E	225 (±42.3)	892 (±92.8)	22 (±3.24)	39 (±10.8)

n/a = not applicable. ^1^ data from Stappaerts et al. [4].

## Data Availability

Data are contained within the article.
